# Coal and gas outburst prediction based on data augmentation and neuroevolution

**DOI:** 10.1371/journal.pone.0317461

**Published:** 2025-02-20

**Authors:** Wenbing Shi, Ji Huang, Gaoming Yang, Shuzhi Su, Shexiang Jiang

**Affiliations:** 1 School of Computer Science and Engineering, Anhui University of Science and Technology, Huainan, Anhui, China; 2 Fujian Provincial Key Laboratory of Medical Big Data Engineering, Fujian Provincial Hospital, Fuzhou, Fujian, China; 3 Anhui Key Laboratory of Mine Intelligent Equipment and Technology, Anhui University of Science and Technology, Huainan, Anhui, China; University 20 Aout 1955 skikda, Algeria, ALGERIA

## Abstract

Coal and gas outburst (CGO) is a complicated natural disaster in underground coal mine production. In constructing smart mines, predicting CGO risks efficiently and accurately is necessary. This paper proposes a CGO risk prediction method based on data augmentation and a neuroevolution algorithm, denoted as ANEAT. First, sample features are applied to the transfer function using a pointwise intensity transformation to obtain new feature samples. It solves the problems of imbalanced data samples and insufficient diversity. Second, the feature importance score sorting and Sparse PCA dimensionality reduction are performed on the data-augmented samples. It provides the initial genome code for the evolutionary neural network. Finally, an evolutionary neural network for CGO prediction is constructed through population initialization, fitness evaluation, species differentiation, genome mutation, and recombination. The optimal phenotype is obtained in the evolutionary generations. In the experiment, we verify the effectiveness of ANEAT from multiple aspects such as data augmentation effectiveness analysis, deep learning model comparison, swarm intelligence optimization algorithm comparison, and other method comparisons. The results show that the *MAE*, *RMSE*, and *EVAR* indexes of ANEAT on the test set are 0.0816, 0.1322, and 0.8972, respectively. It has the optimal CGO prediction effect. ANEAT realizes the high-precision mapping of feature parameters and outburst risk with a lightweight network architecture, which can be well applied to CGO prediction.

## 1 Introduction

Coal and gas outburst (CGO) is a phenomenon in which a large amount of broken coal and gas is suddenly ejected from the coal body to the mining space under geostress and gas joint action. CGO is a very complex natural disaster in the underground production of coal mines, which seriously threatens the production safety of coal mines [1]. China is one of the countries with the most severe outbursts in the world, with more than 600 outburst mines. In recent years, the safety production of coal mines in various countries has improved in detail, but outburst accidents still occur occasionally [2, 3]. Therefore, analyzing and predicting the CGO risk is significant.

The CGO prediction is an intelligent technology that extracts the feature parameters of the coal mining face for risk perception, and its essence is to calculate the collected typical data intelligently. At present, integrating intelligent technology in coal mining and accelerating the construction of smart mines are effective means to reduce accidents, and they are also the only way to promote the safe, rapid, and high-quality development of the coal industry [4, 5]. Regarding research on the mechanism of CGO, domestic and foreign scholars have explored and analyzed the factors, processes, and causes of outbursts. It provides a theoretical basis for predicting, warning, and preventing coal mine outburst disasters [6, 7]. However, how to efficiently analyze and calculate the feature data of the coal mining face and realize high-precision CGO risk prediction has become the focus of current research. Regarding CGO risk prediction and analysis, scholars have put forward corresponding indicators to predict the outburst risk by comprehensively considering the physical and mechanical characteristics of geostress, gas, and coal [8, 9]. This research requires a lot of actual measurement and statistics. It mainly explores the relationship between different indicators and coal seam outburst sensitivity and critical value. In addition, some scholars have analyzed the features of historical CGO events and constructed machine-learning algorithms to predict gas outburst accidents [10, 11]. However, this method usually only optimizes the weights of the network model, not the network structure. When the mining work is in progress, various sensors can also be used to collect coal fracture information and gas change information caused by stress disturbance. It can judge the breeding state of outbursts and give an alarm before the outburst accident is triggered. Scholars have researched outburst feature analysis and early warning regarding acoustic emission, electromagnetic radiation, and microseismic [12–14]. However, in these research methods, acoustic emission monitoring requires a good coupling between the sensor and the coal rock, and electromagnetic radiation monitoring and microseismic monitoring are subject to the complex geographical environment of the mine and signal interference from background noise. The CGO mechanism is complicated, and the outburst risk prediction is a multi-dimensional, complex nonlinear analysis system. Moreover, the CGO accident is very uncertain. It isn’t easy to obtain the features at the time of occurrence, and only some feature parameters after occurrence can be collected. It has the characteristics of numerous outburst-causing factors, insufficient data samples, and imbalanced sample distribution. Due to the complexity of coal seam occurrence conditions and mining environment, the accuracy of outburst prediction still needs improvement. We can observe that existing methods perform poorly in handling imbalanced small sample data and designing model structures.

**Fig 1:**
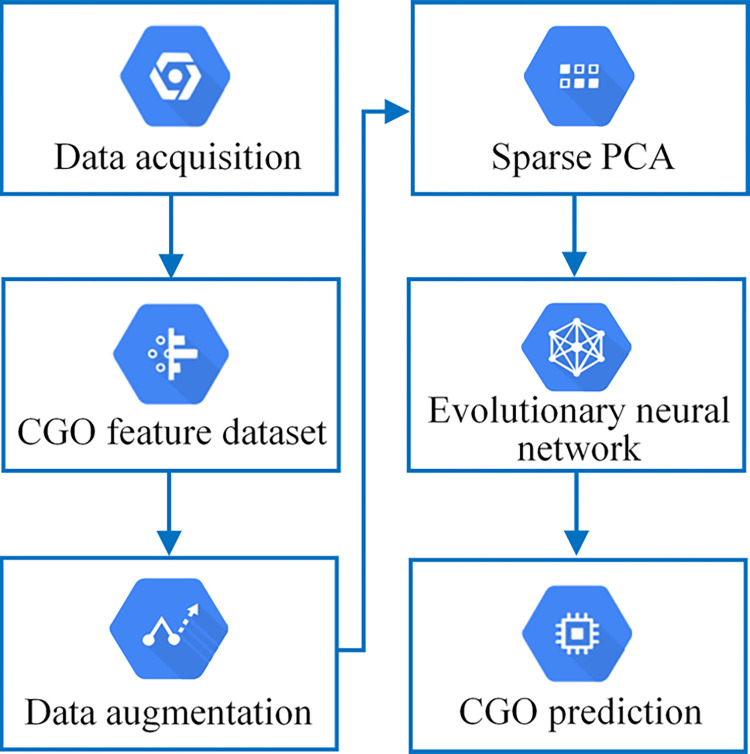
The CGO risk prediction process based on data augmentation and neuroevolution.

To remedy the above limitations, we propose a data augmentation method of pointwise intensity transformation and Sparse PCA to enrich data samples and improve the expression ability of factors leading to CGO. At the same time, we construct an evolutionary neural network to realize the CGO prediction. The model’s structure and weight are trained through the neuroevolution algorithm, thereby improving the accuracy of CGO prediction. The prediction process of CGO risk based on data augmentation and neuroevolution is shown in Fig 1. After the collected data are preprocessed, the original CGO feature dataset can be obtained. Then, data augmentation and Sparse PCA are performed to complete sample reconstruction and feature extraction. Finally, an evolutionary neural network is constructed, and the optimal model is obtained through the evolution of population species to predict CGO risk. In addition, we evaluate the performance of the method by experimental comparison.

The innovation and contribution of this research are as follows:

1. We propose a data augmentation method of pointwise intensity transformation and Sparse PCA tailored to the characteristics of CGO data samples. This method enhances the representation ability of CGO parameters by reconstructing samples and parameters.
2. We construct a CGO prediction method that collaboratively optimizes the model structure and weights by simulating the natural evolution of population species. It breaks through the constraints of traditional machine learning or deep learning preset model architectures and improves the potential of model optimization.
3. We provide a lightweight network architecture for CGO prediction, which achieves high-precision mapping of feature parameters and outburst risk.

The rest of this paper is organized as follows: In Section 2, we give a literature review. Section 3 presents CGO data augmentation and evolutionary neural network construction. Section 4 is presented as an experimental part. Finally, a discussion and conclusion are made in Section 5 and Section 6.

## 2 Literature review

In recent years, intelligent information processing and computing have made significant progress in smart mines. In traditional machine learning, BP can realize complex relationship mapping from input to output, but there may be problems with local optimality and low convergence efficiency during the training process. Wu et al. (2020) proposed a CGO prediction model based on the GASA-BP algorithm. It is a BP model that combines a genetic algorithm (GA) and a simulated annealing algorithm (SA). Their main contribution is to optimize the initial weights and thresholds of BP neural networks with more robust spatial search capabilities [15]. Feature selection is a data preprocessing method that maximizes relevance and minimizes redundancy, enabling efficient data reduction. Zhang et al. (2021) proposed a CGO prediction method integrating feature selection and intelligent optimization classifiers. They constructed the optimal feature subset and sample data through the Boruta and Aprior methods and then used BO-SVM to achieve kernel parameter optimization and prediction classification [16]. Unlike SVM, RVM avoids the limitations of SVM kernel function calculation to a certain extent. Liu et al. (2021) proposed a CGO prediction model named BO-MKRVM. The model combines a Bayesian optimization algorithm and a hybrid kernel RVM. Compared with MKRVM, it has a better fitting effect and generalization ability [17]. To further optimize the predictive performance of CGO, some scholars have adopted a hybrid feature extraction and QPSO-optimized DELM for CGO prediction [18]. Combining DWT, FICA, and LDA feature extraction methods can better eliminate noise features and capture essential information. In addition, the random forest and XGBoost model optimized based on grid search and metaheuristic algorithm have also been applied to gas explosion and CGO prediction [19, 20].

Deep learning has a more complex structure and deeper network layers than traditional machine learning. It can automatically learn more abstract and intricate features. In recent years, CNN has been widely used in smart mining applications. It offers the benefits of local dependence and scale invariance, allowing it to learn hierarchical representations of data automatically [21]. Huang et al. (2018) used CNN to design a method for detecting micro-seismic events in underground mines, which can accurately identify micro-seismic sources [22]. CNN is usually aimed at classification problems but has limitations in target detection. Fast R-CNN introduces the Region Proposal Network on CNN, which can simultaneously perform target detection and classification in the same network. Liu et al. (2020) proposed a rock types intelligent identification method based on Fast R-CNN, which can realize single-type and multi-type rock hybrid image identification. This method is of great significance for evaluating rock mass stability and formulating support plans in mining [23]. With the increase in mining depth and intensity, rockburst poses an increasingly severe threat to coal mine safety production. Zhang (2022) improved Fast R-CNN and built a rockburst prediction model. The improvement of the model mainly includes two aspects: the replacement of Fast R-CNN’s common feature extraction network and the fusion of a probabilistic neural network [24]. LSTM has better applicability when dealing with features with temporal correlation. For dust pollution monitoring in open-pit mines, Li et al. (2021) proposed a hybrid model based on LSTM and attention mechanism to predict total suspended particulate concentration [25]. In gas concentration prediction, some scholars have proposed an innovative Pearson-LSTM model, using the Pearson coefficient to select gas concentration features and LSTM to predict the time series [26]. In coal mine monitoring systems, sensors have complex spatial correlation and temporal variability, and conventional recurrent neural networks cannot adequately capture representative spatiotemporal features. Gao et al. (2023) proposed an attention-based spatiotemporal encoder-decoder network [27]. This method achieves accurate prediction of methane concentration in coal mines through dynamic spatiotemporal dependency learning using a multi-attention mechanism.

In addition to using traditional machine learning and deep learning methods, many scholars have also used other methods to research coal mine risk control, such as teaching learning based optimization, extension theory, and discriminant analysis. Fattahi et al. (2024) used teaching learning based optimization (TLBO) method to predict rock drilling ability in mines. This study can be used to predict rock drilling efficiency and prevent mining accidents [28]. Extension theory is a way to study the possibility, laws, and methods of things expanding through formal models. Wang et al. (2021) built a CGO prediction model based on the extension theory to represent the correlation degree of risk grades quantitatively [29]. Discriminant analysis is a statistical method that can be used for dimensionality reduction or classification [30]. Chen et al. (2020) selected six indicators to construct a multi-indicator comprehensive prediction model for CGO risk based on Bayes discriminant analysis. This method has broad application prospects in CGO risk prediction [31].

Among the above three types of methods, the traditional methods still need to explore optimization and innovative technologies in new application scenarios. The high-precision calculation of deep learning depends on the complex and deep network structure. Other related methods are subject to specific scenarios and applicability. In this research, we mainly focus on the problem of insufficient data samples and imbalanced sample distribution. We also explore a lightweight CGO prediction method that collaboratively optimizes the model structure and weights.

## 3 CGO data augmentation and evolutionary neural network construction

### 3.1 Causing factors and data sources of CGO

CGO is still one of the critical hazards that threaten the safe production of modern mines, and many factors cause CGO. Geological structural characteristics will cause changes in gas storage in coal bodies. The structural condition of the surrounding rock around the coal body will affect the gas content or pressure. Crustal stress factors also play an essential role in CGO. In addition, the physical properties of the coal body, the gas state, the thickness of the coal seam, and mining technology influence CGO accidents.

In this research, the data for CGO prediction come from the data set published in the thesis of China University of Mining and Technology [32]. The data was collected from two mining faces of 3405 and 3406 in a coal mine in Shanxi, China. Data acquisition and processing have contributed to the exploration and research of CGO intelligent prediction. According to the causing factors of CGO and the actual detection situation of the mine site, the feature parameters in the data set include gas characteristics, coal seam characteristics, and geological structure. The parameters of gas characteristics include gas content, initial velocity of gas release, and gas desorption index of drill cuttings K1. Coal seam characteristics include the coal type of damage, soundness coefficient of coal, burial depth, coal thickness, and cuttings amount. The geological structure characteristic is mainly the distance from the geological tectonic zone. According to the actual situation at the site, no safety measures have been taken, and it is considered that there is no danger of CGO. A value of 0.1 indicates the risk is very low or relatively safe. Drilling 12–15 groups of pressure relief drilling holes means that the CGO risk is average, and the risk value is represented by 0.6. Drilling 20 groups of pressure relief drilling represents a severe risk. A severe risk is indicated by a value of 1.0. The CGO sample feature can be defined as Eq 1, and the CGO risk value corresponding to the data sample is defined in Eq 2.


$$gof=[\begin{array}{cccc} x_{11} & x_{12} & \ldots & x_{1n}\\ x_{21} & x_{22} & \ldots & x_{2n}\\ \ldots & \ldots & \ldots & \ldots \\ x_{m1} & x_{m2} & \ldots & x_{mn} \end{array}]$$ (1)

$$rv=[\begin{array}{cccc} v_{1} & v_{2} & \ldots & v_{m} \end{array}]$$ (2)

### 3.2 Sample data augmentation

CGO results from many factors, and its risk prediction is a typical multi-factor nonlinear problem. CGO is a small probability event, and collecting real-time data on outburst occurrence is difficult. In the actual detection of coal mines, the overall data obtained is small, and the samples are imbalanced, which belongs to small sample data. These actual conditions have brought challenges to the accurate prediction of CGO risk. Data augmentation is artificially augmenting data to generate new samples based on existing data. It can increase the number of samples and enrich the diversity of features.

**Fig 2:**
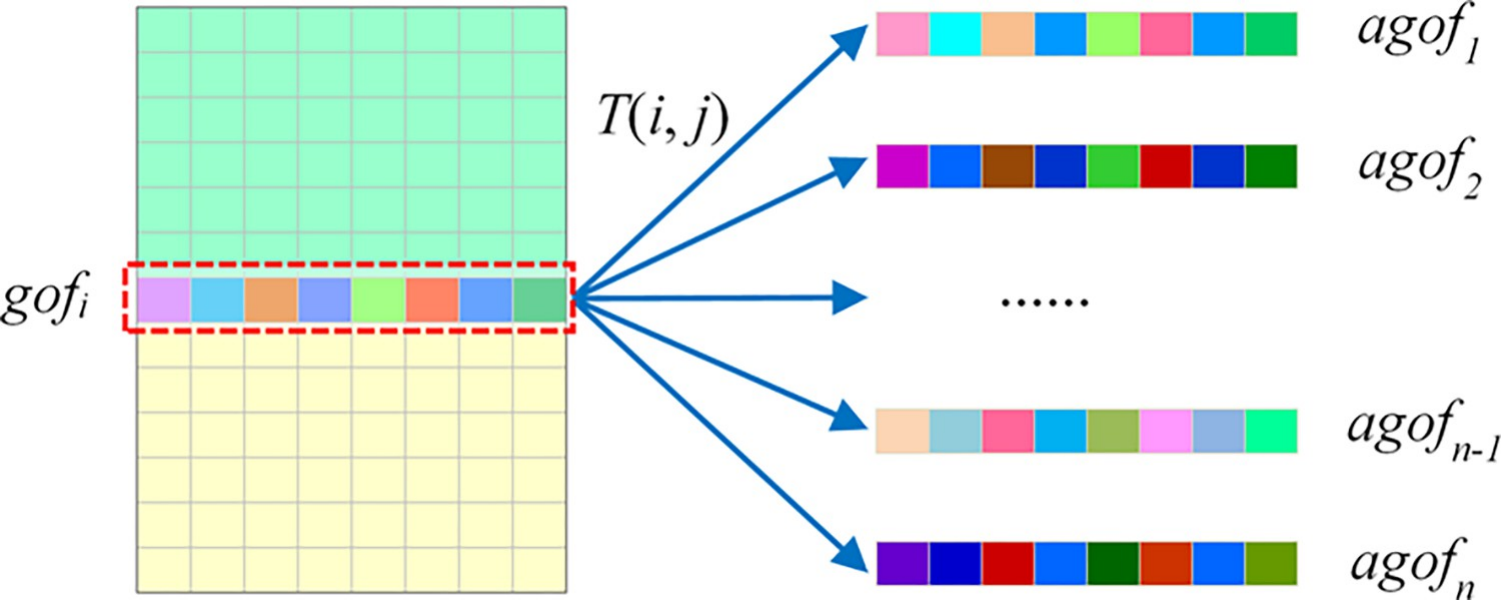
The principle of sample data augmentation.

This research proposes a data augmentation method of pointwise intensity transformation. It applies sample features to the transfer function *T*(*i*, *j*) and constructs the generated output values as new feature samples. The transfer function *T* is shown in Eq 3, where *rand*_*i*_(*c*_1_,*c*_2_) is a random value between *c*_1_ and *c*_2_ generated for the *i*-th sample, and *gof*[*i*, *j*] is the feature value in the sample. The transformed output intensity depends on the original sample feature values. After the feature values of the same sample are applied to the transfer function *T*(*i*, *j*) multiple times, the number and diversity of samples will be increased. The principle of data augmentation for CGO feature samples is shown in Fig 2. The *gof*_*i*_ in the figure is the *i*-th feature sample in the original data. After *gof*_*i*_ is continuously applied to the transfer function *T*(*i*, *j*) *n* times, the augmented feature samples *agof*_*1*_ to *agof*_*n*_ will be obtained.


$$T(i,j)=rand_{i}(c_{1},c_{2})*\log(1+gof[i,j])$$ (3)

### 3.3 Construction of evolutionary neural network model

The evolutionary neural network is an important branch of artificial neural networks inspired by natural evolution. The basic structure of the traditional artificial neural network is fixed, and the structure remains unchanged during the training process. Only neurons’ biases and connection weights are continuously optimized [33]. The mechanism of CGO is complicated, and there is a complex nonlinear relationship among the multi-parameters of outburst risk. The traditional artificial neural network has problems such as long convergence time and easily falling into a locally optimal solution. This study proposes a CGO prediction model based on the evolutionary neural network. The optimal network is gradually constructed through the survival of the fittest. Model evolution starts with a small, simple population of genomes and progressively increases in complexity over generations. The weight and topology of the network are continuously optimized to obtain the optimal network.

#### 3.3.1 Genome coding

**Fig 3:**
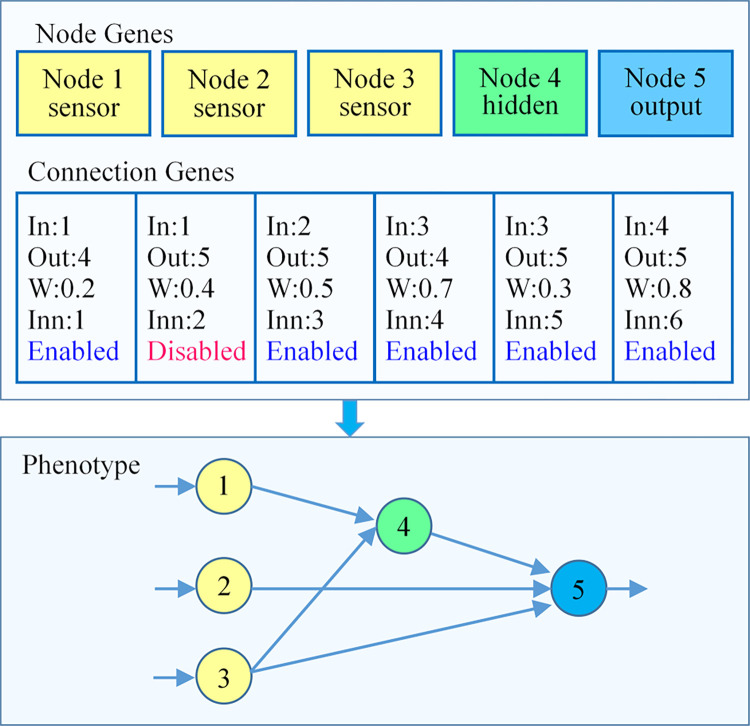
The genome coding scheme.

An essential work in CGO prediction model construction is the genomic representation of evolutionary neural networks. In this research, we adopted the approach of direct genome encoding. The genome code is composed of linker genes and node genes. The node gene provides the list information of the input, hidden, and output layer nodes in the artificial neural network. The connection gene provides list information such as connection weight, innovation number, and enabled status between nodes. In addition, the node’s initial bias value is generated by system initialization. The genome encoding scheme of the neuroevolution algorithm is shown in Fig 3. There are five elements in the node gene list and six in the connection gene list. The neural network in the figure is the phenotype corresponding to the genome coding.

#### 3.3.2 Structural mutation

**Fig 4:**
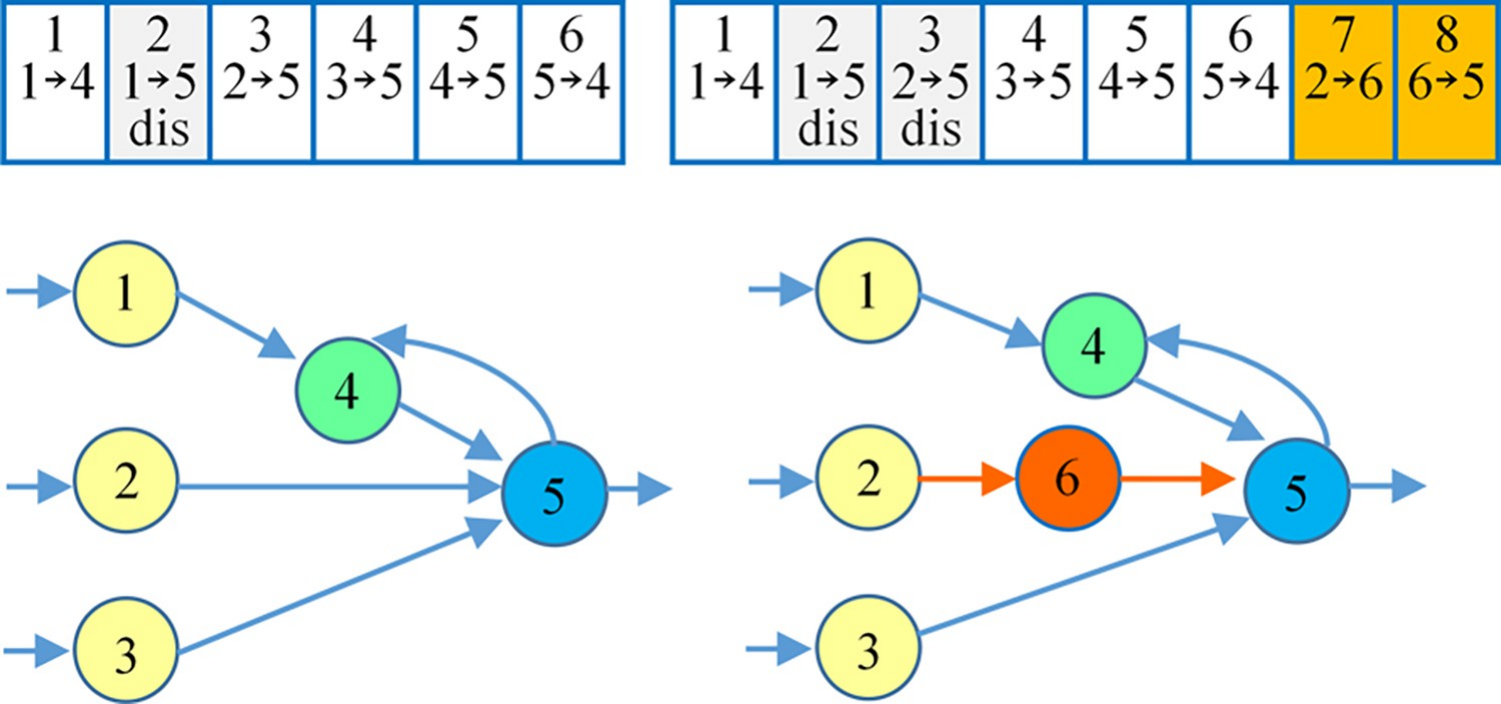
The structural mutation of neuroevolution.

Mutation refers to the difference in some traits between the offspring and the parents of the organism, as well as between different individuals of the offspring. Whether the mutation is beneficial to the organism can be divided into favorable mutation and unfavorable mutation. Favorable mutations that are heritable give rise to new biotypes. It enables organisms to evolve from simple to complex, from low to high. This mutation makes the organism better adapted to the environment in which it lives. The evolutionary neural network’s mutation operator can change the neural network’s connection weight and structure. Structural mutation mainly includes adding new connections and adding new nodes. When the mutation operator is applied to the genome, newly added link genes or node genes will be assigned an increasing innovation number. An example of structural mutation is shown in Fig 4. New node six is added to a specific location in the genome, and the original weighted interconnection in the neural network is split and disabled to adapt to the new node. Then, two new genes with innovation numbers 7 and 8 are added at the end of the genome.

#### 3.3.3 Genome recombination

**Fig 5:**
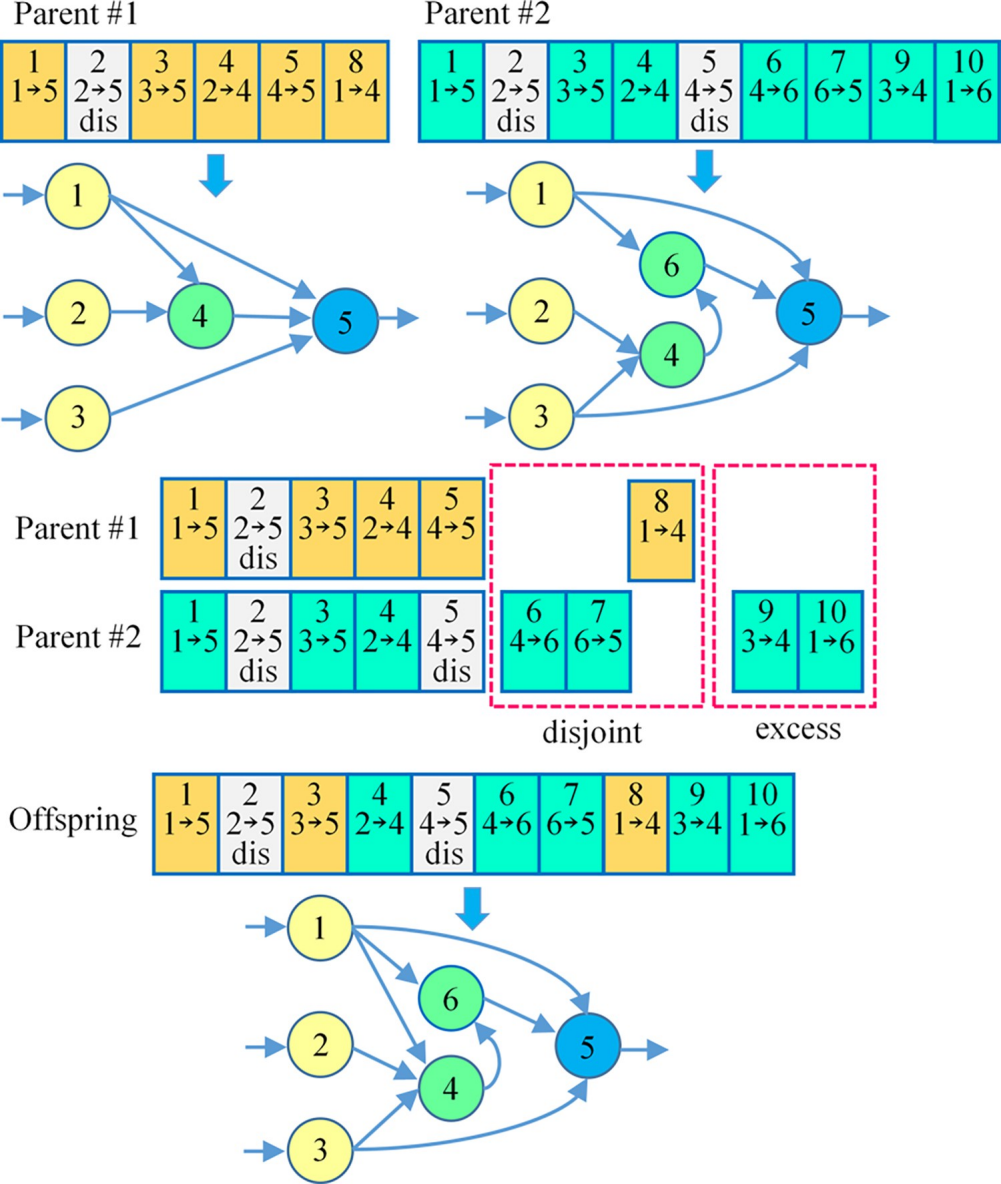
The genome recombination of neuroevolution.

Genome recombination is also known as crossover. It is the DNA at the same position of two chromosomes is cut off, and the two strands before and after are crossed and combined to form a new chromosome. Genome recombination aims to make the parents’ genes match together, create characteristics that the parents did not have, generate new and more favorable genotypes, and improve the viability of offspring. When two genomes are structurally crossed, the two parental genomes are first paired according to the innovation number, and the genes with the same innovation number are aligned sequentially. If the innovation numbers do not match, the gene belongs to a disjoint or redundant section of the genome, and these mismatching innovation numbers will be lined up separately. The genome recombination process in neuroevolution is shown in Fig 5. The genes of innovation numbers 1–5 in parent one and parent two are matched, and the genes of one side are randomly selected to produce offspring. In addition, innovative numbers 6, 7, and 8 are disjoint parts, and innovative numbers 9 and 10 are redundant parts. Disjoint and redundant genes are added unconditionally from either parent and ranked by innovation number. Thus, the offspring genome and corresponding neural network are obtained, as shown in Fig 5.

#### 3.3.4 CGO prediction model construction based on neuroevolution

The speciation method is the key to the CGO prediction model based on neuroevolution. It will guide species to innovate various topological forms successively from generation to generation and promote the diversity and innovation of species. Speciation differentiates a population into different subspecies or species. It imposes a specific differentiation and isolation mechanism by calculating the difference distance, encourages the exploration and evolution within each subgroup, and avoids prematurely falling into a locally optimal solution. The calculation of the difference distance *δ* between individuals is shown in Eq 4, where *k*_*1*_, *k*_*2*_, and *k*_*3*_ are weight coefficients, *N* is the number of genes on the side with the larger number of genes in the comparison individual, and *D* is the number of disjoint genes when matching, *E* is the number of redundant genes when matching, and $\bar{W}$ is the average difference of the weights of two individual neural networks. When *δ* is less than the set threshold, the compared individuals are regarded as the same species. When the *δ* values calculated by an individual for all other species are higher than the set threshold, it is considered the representative individual of the new species.


$$\delta =\frac{k_{1}D}{N}+\frac{k_{2}E}{N}+k_{3}\bar{W}$$ (4)

In constructing the CGO prediction model, the fitness of the evolutionary individual reflects the quality of the risk prediction effect. Individuals with high fitness will have a higher probability of continuing to evolve in the next generation, while individuals with low fitness will be eliminated more easily. The calculation method of fitness is shown in Eq 5, where *F*_*init*_ is the initial constant value, $rv_{i}^{\prime }$ and *rv*_*i*_ are the predicted and actual values of CGO risk, respectively.


$$fitness_{i}=F_{init}-\sum_{i=1}^{n}\sqrt{{\left(rv_{i}^{\prime }-rv_{i}\right)}^{2}}$$ (5)

The individual’s fitness determines the reproductive probability during the model’s evolution. In order to prevent individuals with high fitness from gradually replacing the entire population due to increasing numbers, the CGO prediction model performs fitness corrections before the species reproduces and evolves. The adjusted fitness calculation method is shown in Eq 6; *n* is the number of individuals in each generation, and *δ*(*i*, *j*) is the difference distance between individuals *i* and *j*. In addition, *threshold*(*δ*(*i*, *j*), *T*) is a threshold function; when *δ*(*i*, *j*) is higher than the threshold *T*, the return value is 0; otherwise, the return value is 1. The fitness adjustment method can better inhibit the rapid expansion of mature populations, stimulate the diversity of species evolution, and play a vital role in the evolution of the model.


$$adjfitness_{i}=\frac{fitness_{i}}{\sum_{j=1}^{n}threshold(\delta (i,j),T)}$$ (6)

**Fig 6:**
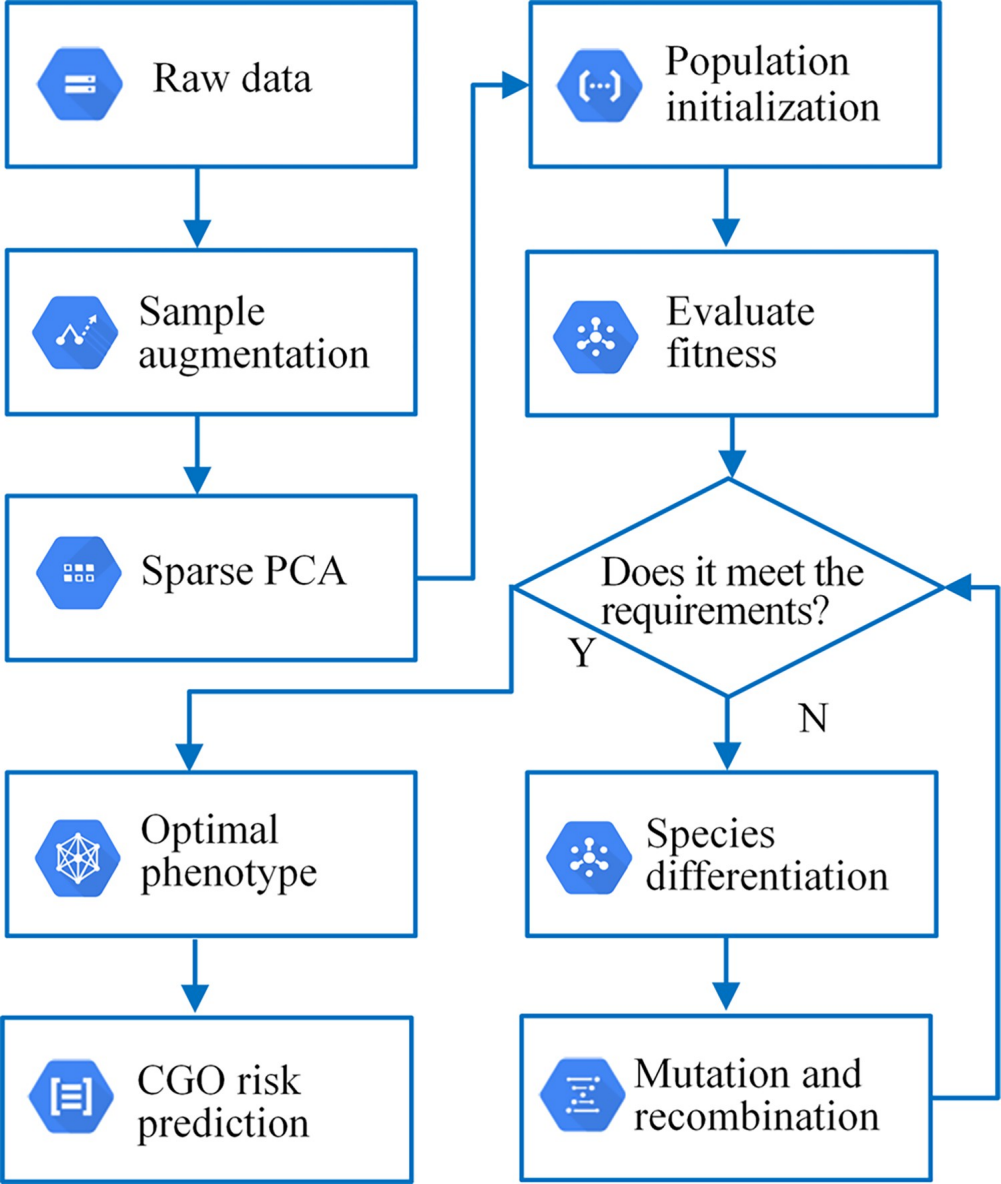
The construction process of the CGO prediction model.

The construction process of the CGO prediction model is shown in Fig 6. The raw data has been enhanced in representation ability through sample augmentation, and then the dimensionality is reduced by Sparse PCA. The principal components of CGO features are highlighted. The evolution of the model is guided by optimization. Based on the above work, the evolutionary neural network is initialized to allow the model to evolve from the initial simple topology, and the population’s average fitness and maximum fitness are calculated. Suppose the model evolution does not reach the maximum number of generations or is less than the fitness threshold. In that case, the population is differentiated by adjusting the fitness, and then the model is evolved according to genome mutation and recombination. The optimal phenotype is taken as the final model if the network evolution meets the set requirements.

## 4 Experiment and analysis

This research proposes a CGO prediction method based on data augmentation and an evolutionary neural network for high-precision risk prediction with lightweight structures. We preprocessed the raw data and performed sample data augmentation in the experiment. In the augmented data sample, Sparse PCA dimensionality reduction is performed by calculating the feature contribution rate to avoid invalid hidden nodes or connections in the evolutionary structure of the network model. On the basis of these works, the optimal phenotypes are evolved through population initialization, fitness evaluation, species differentiation, genome mutation, and recombination. In order to verify the effectiveness of this method, we analyzed and compared the effect of CGO prediction in data augmentation and non-data augmentation modes. The data-augmented evolutionary neural network is described as ANEAT, and the non-augmented evolutionary neural network is described as NANEAT. In addition, MLP, XGBoost, and PSO-SVR models are also selected for comparative experiments.

This experiment runs on Windows 10 Professional Edition 64-bit operating system, Intel i9-13900F CPU, and 32G RAM. In addition, libraries such as Sklearn, Pytorch, and NEAT-Python are also adopted in the experiment.

### 4.1 Data preprocessing

The experimental data come from two mining faces of 3405 and 3406 in a coal mine in Shanxi, China, and the feature parameters include gas characteristics, coal seam characteristics, and geological structure. The structure of the sample is shown in Table 1. The CGO features are non-time series, and the extremum method is used for dimensionless processing in the experiment. The calculation of extreme value is shown in Eq 7, where *x* is the original value of the feature, *x*_*max*_ and *x*_*min*_ are the maximum and minimum values of the feature, and *x*’ denotes the value after dimensionless processing.


$$x^{\prime }=\frac{x-x_{\min}}{x_{\max}-x_{\min}}$$ (7)

**Table 1:** The structure of the data sample.

Parameter	Description
tcd	Types of coal damage
ivgr	The initial velocity of a gas release
scc	The soundness coefficient of coal
csgc	Coal seam gas content
k1gdc	Gas desorption index of drill cuttings k1
ca	Cuttings amount
dgtz	Distance from geological tectonic zone
bd	Buried depth
ct	Coal thickness
gor	Gas outburst risk

In the experimental data, a CGO risk value of 0.1 indicates that the risk is very low or relatively safe, 0.6 indicates a moderate risk, and 1.0 indicates an extremely high risk. Because there are few samples with a risk value of 1.0 in the original data, and the sample distribution is highly imbalanced. In the experiment, we applied the sample feature *gof*_*i*_ with a risk value 1.0 to the pointwise intensity transfer function *T*(*i*, *j*) three times in a row and obtained the corresponding augmented feature samples *agof*_*1*_, *agof*_*2*,_ and *agof*_*3*_. It increases the number and diversity of samples of this type and constructs an augmented sample dataset. In order to maintain the consistency of the sample data, the samples with risk values of 0.1 and 0.6 were also treated once with a corresponding transfer function.

**Fig 7:**
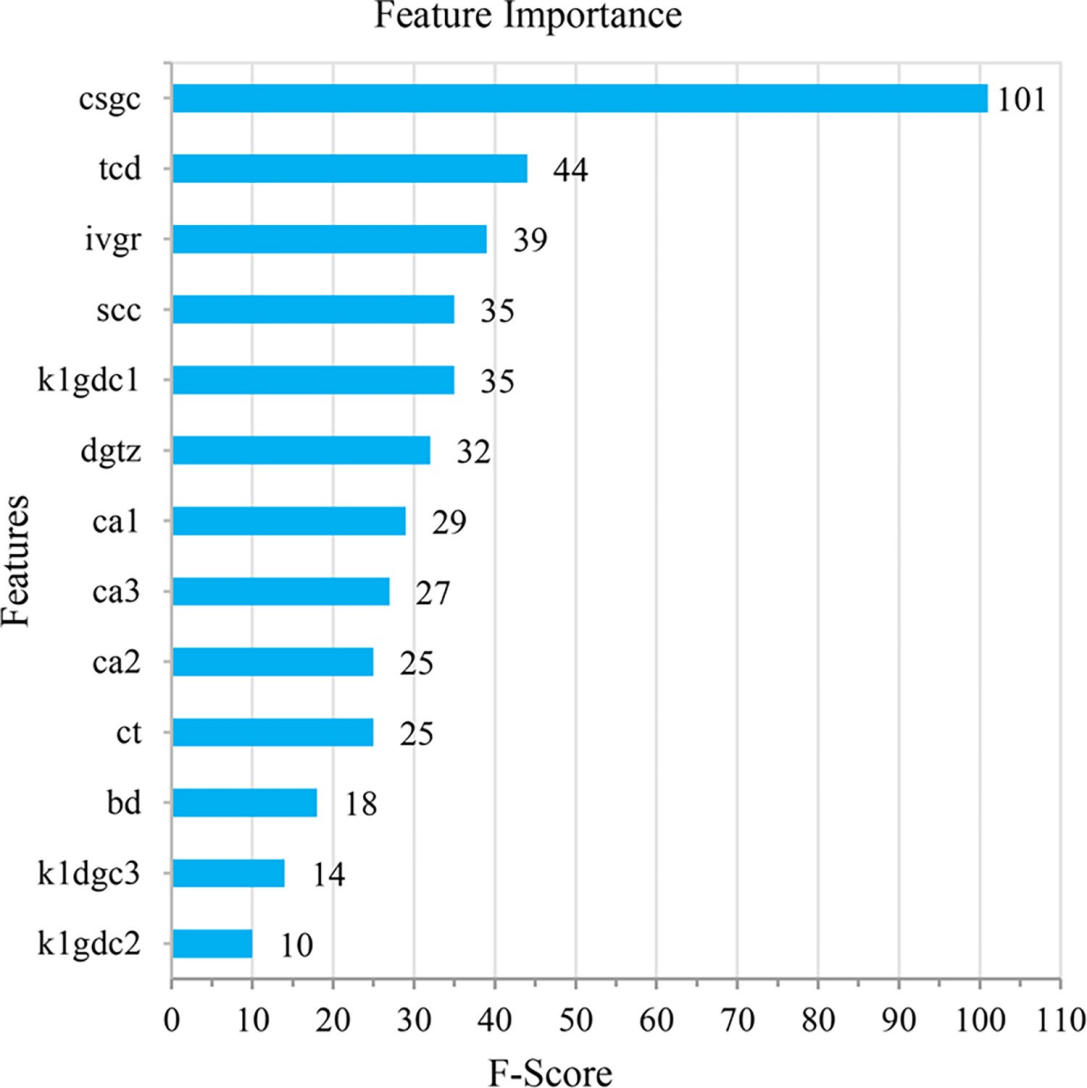
The analysis of feature importance.

The initial genome code of the evolutionary neural network plays an essential guiding role in the evolution of the model. In order to avoid redundant hidden nodes and connections during model evolution, feature importance analysis and Sparse PCA dimensionality reduction were carried out in the experiment. We use XGBRegressor to analyze the importance of the feature for the augmented sample dataset. The F-Score values of each feature are shown in Fig 7.

The results show that the importance contribution rate of the top 10 features is above 90%. Since some features contain 3 component values, the number of principal components of Sparse PCA is finally set to 8. After the original data is subjected to extreme value calculation, sample feature augmentation, feature importance analysis, and Sparse PCA dimensionality reduction, the obtained data sample includes eight principal component features, denoted as *f1*-*f8*. They constitute the initial genome code of the evolutionary neural network.

### 4.2 The evolution of the CGO prediction model

The input layer of the CGO prediction model includes eight parameters of *f1*-*f8*, and the algorithm generates the nodes and connection structure of the hidden layer through several generations of iterative self-evolution. The model output is a risk value between 0 and 1. To evaluate the effect of CGO prediction, mean absolute error *MAE*, root mean square error *RMSE*, and explicable variance score *EVAR* are used as evaluation indexes. The corresponding calculation methods are shown in Eq 8, Eq 9, and Eq 10, where *y* is the expected value, *y’* is the target value output by the model, and *VAR* represents the calculation variance. Among them, the *EVAR* can reasonably measure the degree of interpretation of the model to the fluctuation of the dataset, and the value interval is [0,1]. If the *EVAR* value is higher, the discrete distribution of the model output value and the sample expected value is closer, and the effect of CGO prediction is better.

$$MAE(y,y^{\prime })=\frac{1}{n}\sum_{i=1}^{n}\left|y_{i}-y_{i}^{\prime }\right|$$ (8)

$$RMSE(y,y^{'})=\sqrt{\frac{1}{n}\sum_{i=1}^{n}(y_{i}-y_{i}^{'}{)}^{2}}$$ (9)

$$EVAR(y,y^{'})=1-\frac{VAR(y-y^{'})}{VAR(y)}$$ (10)

**Fig 8:**
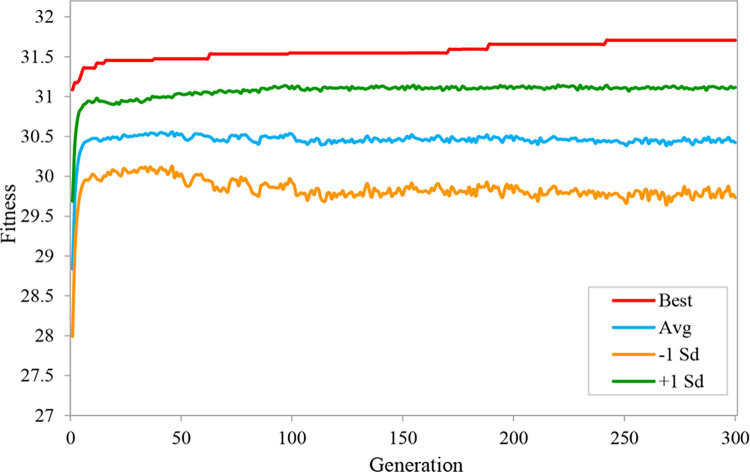
The fitness of ANEAT model evolution.

We built an evolutionary neural network environment with augmented topology based on the Neat-Python 0.92 library in the experiment. We divided the dataset into ten parts, taking turns using nine of them as training data and one as testing data. The experimental results are taken as the average of 10-fold cross validation. The main parameter settings of the evolutionary neural network are shown in Table 2, where fitness_criterion is the standard function for calculating fitness, fitness_threshold represents the threshold for fitness evolution, and pop_size sets the number of organisms involved in evolution in each generation. In model evolution, Eq 5 is used to calculate fitness. After 300 generations of evolution on the augmented dataset, the fitness of ANEAT model evolution is shown in Fig 8. The Best in the figure is the optimal fitness of the species during the evolution process; the Avg denotes the population’s average fitness, the -1 Sd means that the average fitness is reduced by one standard deviation, and the +1 Sd means that the average fitness is plus one standard deviation. The experimental results show that the best fitness of the population species gradually increases to 31.47 from the first generation to the 38th generation, which then tends to be stable and offers a slight upward trend. When evolving to the 242nd to 300th generation, the best fitness increased to a maximum value of 31.71. The average fitness of the population species increased to 30.52 in the 24th generation. It showed a slight fluctuation and slight increase in the subsequent evolutionary process. With the advancement of evolutionary generations, the average fitness increased to 30.56.

**Table 2:** The parameter setting of the evolutionary neural network.

Parameter	Parameter value
fitness_criterion	max
fitness_threshold	35.0
pop_size	1600
activation_default	sigmoid
aggregation_default	sum
compatibility_disjoint_coefficient	1.0
compatibility_weight_coefficient	0.5
compatibility_threshold	3.0
species_fitness_func	max
species_elitism	2

**Fig 9:**
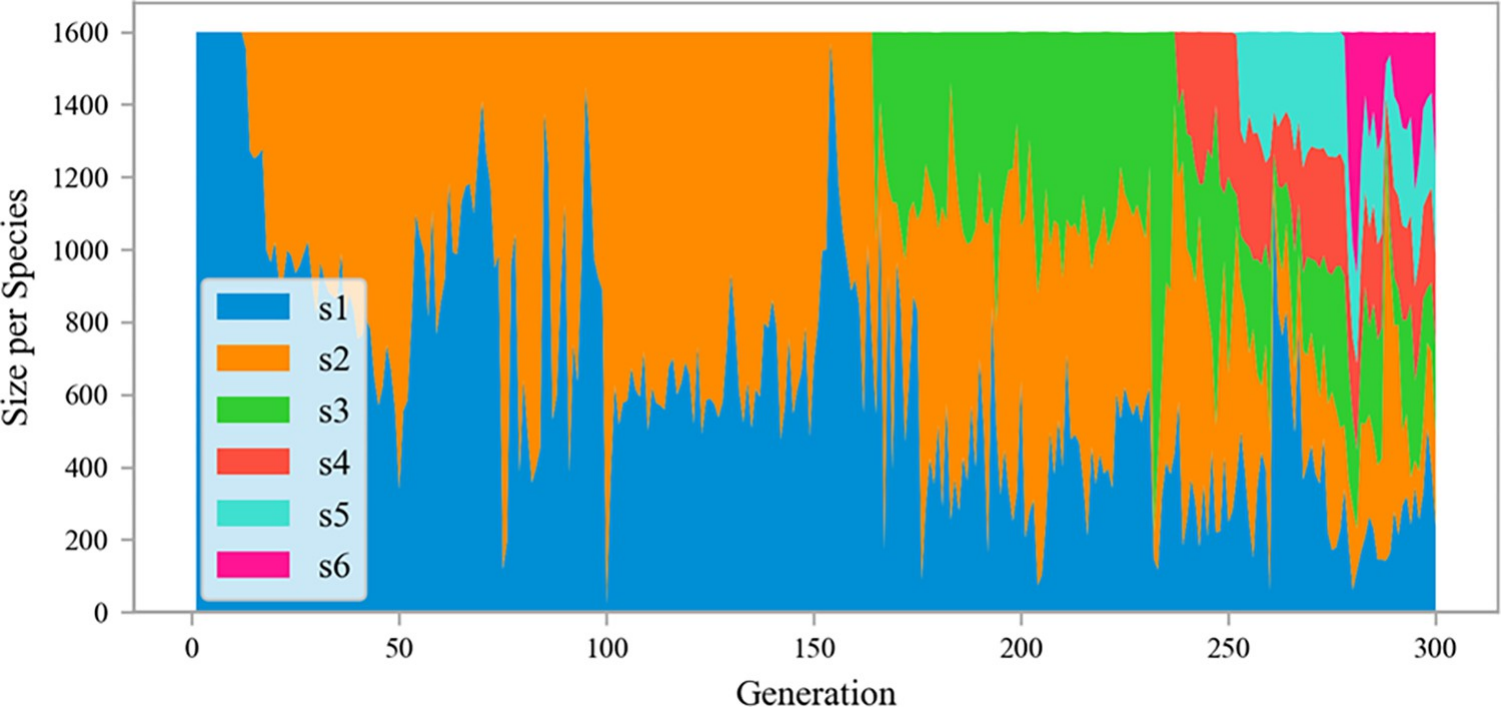
The speciation of ANEAT model evolution.

As the model introduced the function of speciation, certain species exhibited superior performance in early generations and retained beneficial mutations, eventually resulting in champion organisms. Fig 9 is a stacked diagram of population speciation during the evolution of the ANEAT model. It shows how populations of organisms evolve through generations of speciation. The symbols s1-s6 in the figure represent six types of species, and the evolution starts from a single species s1, which occupies the entire population. Species s2 began to germinate in the 13th generation. In the later stage of evolution, the population diverged into four more species in the 165th, 238th, 253rd, and 278th generations, producing champion organisms finally.

**Fig 10:**
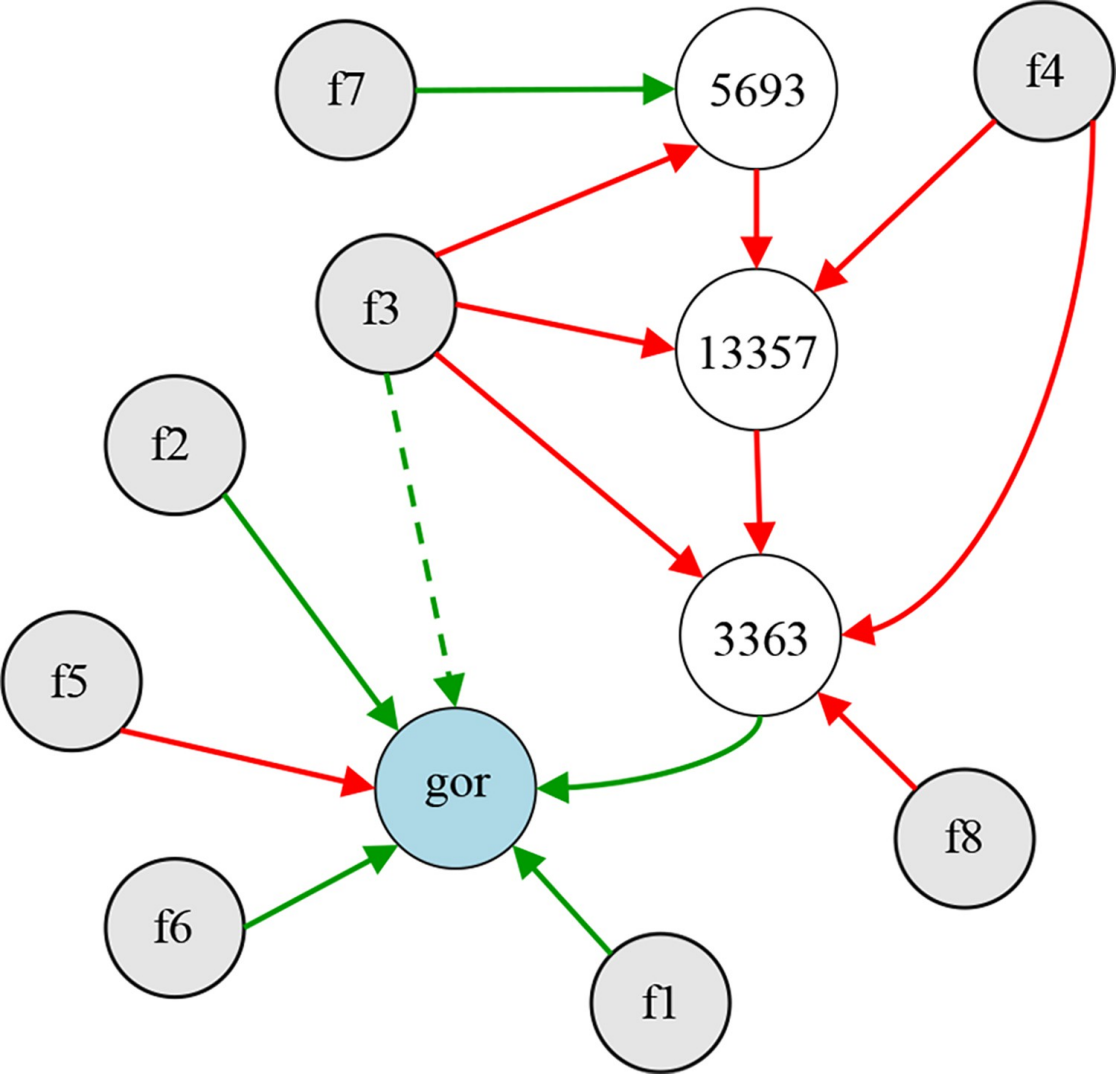
The structure of the ANEAT model corresponds to the optimal species genome.

In the evolution of 300 generations, the species with the best fitness is selected as the champion organism, and the phenotype of its genome is the final ANEAT model. According to the genome code, the network model drawn using python graphviz is shown in Fig 10. It is an ANN phenotype consisting of 12 nodes and 15 connections. The gray nodes *f1*-*f8* in the figure are input nodes, the nodes with id numbers 3363, 5693, and 13357 are hidden nodes, and the symbol gor represents the output node of CGO risk value. Neat-Python does not assign a separate node for bias but assigns a bias attribute value to network nodes, so the bias node is not shown in Fig 10. The red connection in the figure indicates that the weight is less than 0, the green connection indicates that the weight is greater than 0, and the dotted line indicates that the enabled attribute of the connection gene is false. The thickness of the connection reflects the difference in the absolute value of the connection weight. The results show that the evolved ANEAT model is a lightweight ANN structure. It results from the collaborative optimization of network structure and weights and reflects the phenotype of champion organisms in the population.

**Fig 11:**
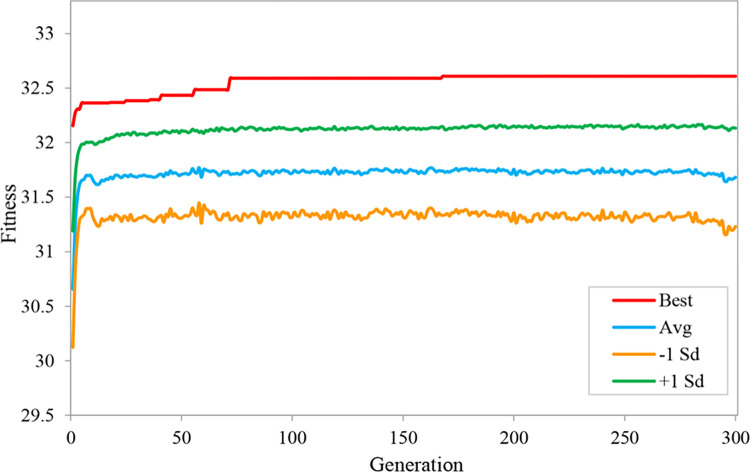
The fitness of NANEAT model evolution.

In order to verify the effectiveness of the data augmentation method in CGO prediction, we also carried out the construction experiment of the NANEAT model on the non-augmented dataset using the same data processing method. During the evolution of 300 generations, the fitness changes of the NANEAT model evolution are shown in Fig 11. The best fitness of the NANEAT model species gradually increased to 32.59 from the first generation to the 72nd generation, and it tended to be stable and slightly improved in the evolution of subsequent generations. The best fitness maintained a stable maximum value of 32.61 from the 168th generation to the 300th generation. The average fitness of population species increased to 31.71 in the 40th generation, then stabilized and slightly rose to 31.77 in the subsequent evolutionary process.

**Fig 12:**
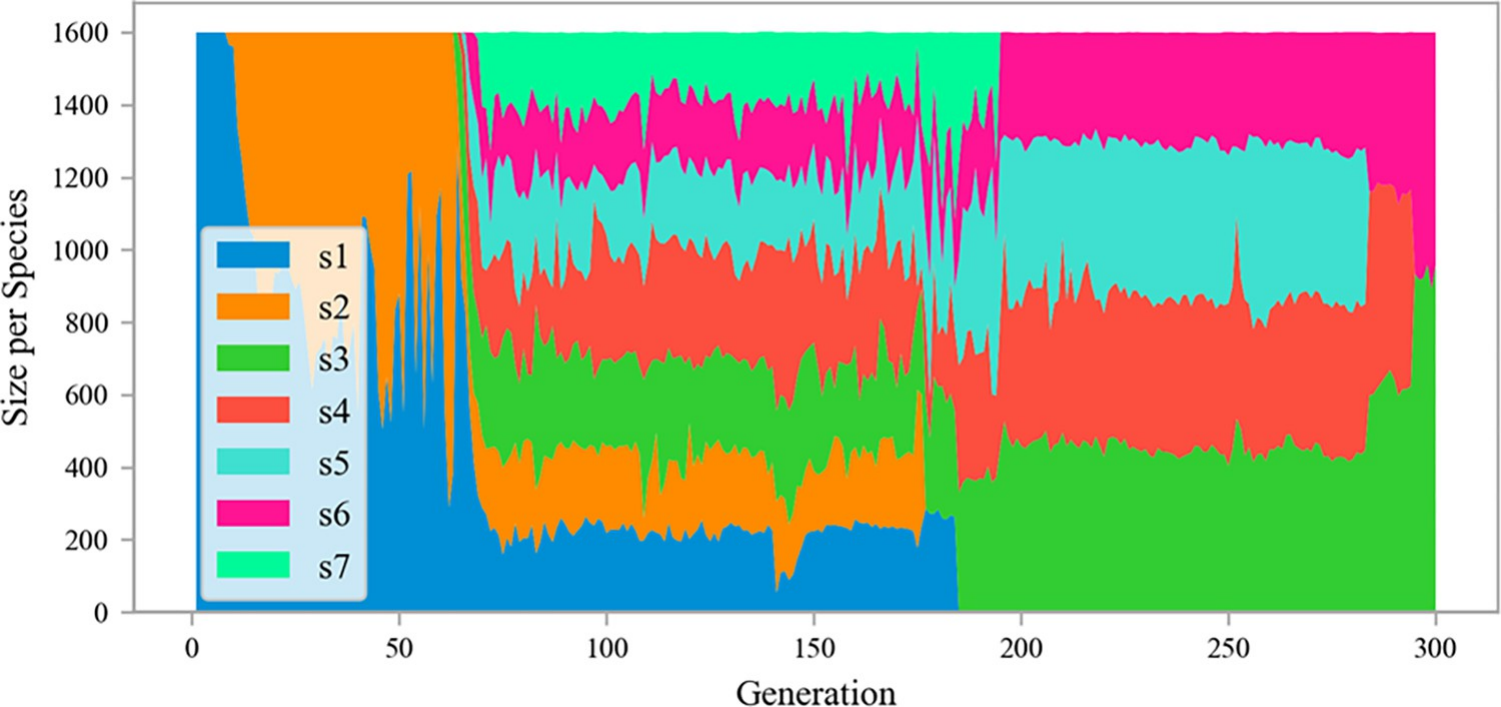
The speciation of NANEAT model evolution.

The speciation of the evolutionary process of the NANEAT model is shown in Fig 12. During the evolution of 300 generations, the population produced seven types of species, s1-s7. The population started with a single species s1. Species s2 began to germinate in the 9th generation, species s3 and s4 germinated in the 64th and 65th generations, respectively, and new species s5 and s6 continued to germinate in the 66th generation. When it evolved to the 69th generation, a new species s7 emerged. With the advancement of evolutionary generations, species s2 disappeared in the 177th generation, s1 in the 185th generation, and s7 in the 195th generation. From generation 196 to generation 284, species s3, s4, s5, and s6 occupied the entire population. When the evolution reaches the 285th generation, species s5 disappears, and the final population contains species s3, s4, and s6.

**Fig 13:**
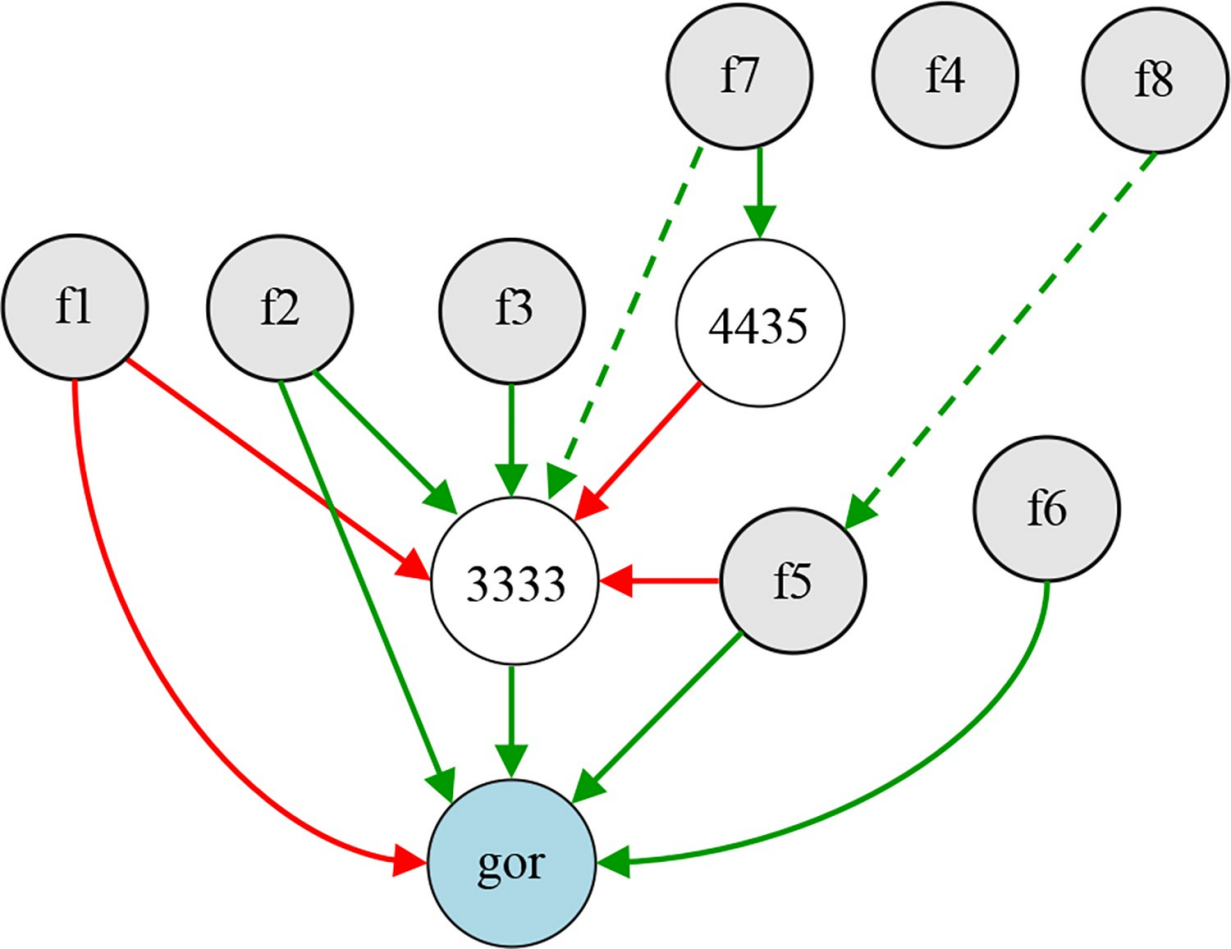
The structure of the NANEAT model corresponds to the optimal species genome.

Using the same selection method as the optimal phenotype of the ANEAT model, the champion organism with the best fitness was selected, and the NANEAT model corresponding to its genome phenotype is shown in Fig 13. The NANEAT model is an ANN consisting of 11 nodes and 13 connections. The enabled attribute of the connection gene corresponding to the two green dotted lines in the figure is false, and the *f4* node has no corresponding connection. In fact, the nodes *f4*, *f7*, and *f8* do not generate data input. Analyzing the phenotype of the NANEAT model, we can see that the optimal fitness of the species in the population did not reach the threshold of 35 during the evolution of 300 generations. The model undergoes structural mutations while maintaining the current optimal fitness, and the model shows a tendency of over-evolution.

Combining fitness changes and population speciation during NANEAT evolution, we found that the best fitness maintained a stable value of 32.61 from the 168th generation to the 300th generation. The best fitness of subsequent evolution has not been improved, and some species in the population have been extinct since the 177th generation. The best fitness is the best stable state between 32.59 and 32.61. During this evolution process, seven types of species maintained a total of 105 generations. In subsequent evolutionary generations, the fitness was not improved, and the species diversity of the population gradually decreased. The final population has only three types of species. From these state changes, it can be seen that the diversity of data samples is not enough to drive the model to evolve in a beneficial direction. Compared with the NANEAT model, the ANEAT model has a more stable evolutionary state and clearer beneficial evolutionary trends. It shows that data augmentation can have a positive effect on the evolution of the model.

In terms of the fitness of model evolution, the best fitness of the ANEAT model is 31.71, and the best fitness of the NANEAT model is 32.61. Since the two models are evolved and trained in independent samples, the value of optimal fitness cannot fully measure the model’s prediction accuracy. The CGO prediction accuracy of the model needs to be evaluated on the same test set in combination with specific indexes.

### 4.3 Comparative analysis of experimental results

In order to verify the effectiveness of the method proposed in this paper, ANEAT and NANEAT models were compared and analyzed. In addition, MLP, XGBoost, and PSO_SVR models are also used for comparative experiments. The essence of the ANEAT model is a lightweight artificial neural network in which the structure and weights evolve collaboratively in a data-augmented environment, while the MLP is a multi-layer fully connected neural network. Comparative experiments can reflect the prediction effect of multi-layer neural networks and evolutionary neural networks. In addition, the XGBoost method using grid search is also chosen for comparative analysis [20]. In terms of the swarm intelligence optimization algorithm, the PSO_SVR method is used for comparative analysis.

Similarly, using 10-fold cross validation, the experimental results of each model on the test set are shown in Table 3. In addition to the mean absolute error (*MAE*) and root mean square error (*RMSE*), the evaluation indexes also include the explicable variance (*EVAR*). The *EVAR* is located in the value interval [0,1], which reflects the discrete distribution of predicted and expected values. A higher *EVAR* indicates that the discrete distribution of the predicted values is closer to the expected value, signifying improved predictive performance of the CGO model. Among them, ANEAT has the lowest *MAE* (0.0816) and *RMSE* (0.1322) value, and *EVAR* (0.8972) is the highest, which has the best prediction effect. Its *EVAR* is 0.3183 higher than MLP and 0.0718 higher than XGBoost. In terms of the swarm intelligence optimization algorithm, both ANEAT and NANEAT are better than PSO_SVR. In terms of data augmentation, the *MAE* and *RMSE* of ANEAT are lower than those of NANEAT, and the *EVAR* of ANEAT is 0.0859 higher than that of NANEAT. The results show that the predictive performance of ANEAT is superior to other comparative models. It demonstrates the effectiveness of evolutionary neural networks and data augmentation.

**Table 3:** Evaluation indexes of different methods on the test set.

Model	MAE	RMSE	EVAR
MLP	0.2907	0.3417	0.5789
XGBoost	0.0832	0.1708	0.8254
PSO_SVR	0.1636	0.1820	0.7895
NANEAT	0.0951	0.1701	0.8113
ANEAT	0.0816	0.1322	0.8972

**Fig 14:**
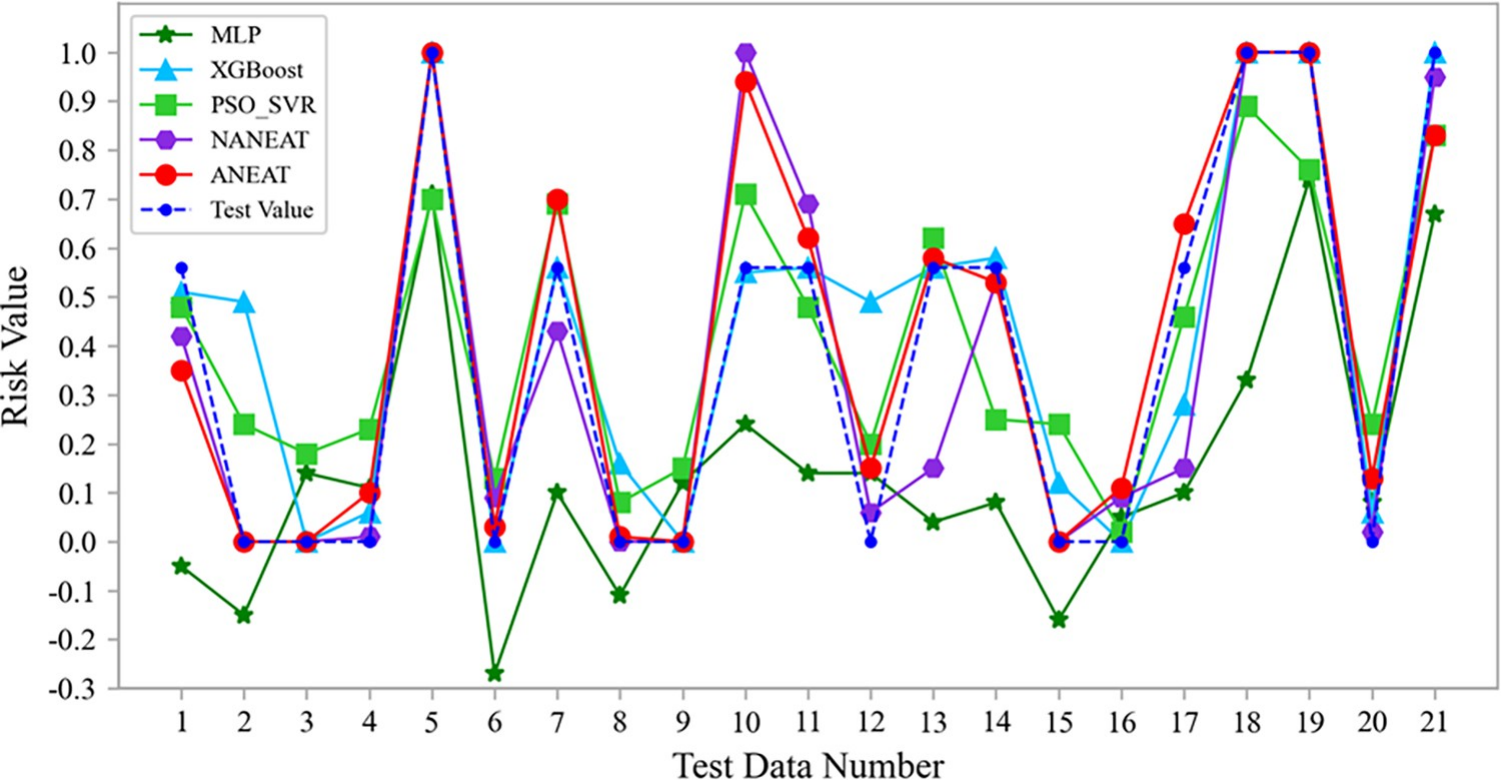
The comparison of different prediction methods on the test set.

To compare and analyze the predictive performance of each model on CGO risk more intuitively, we draw the line charts of the ANEAT model and other models on the test samples. The comparison of test results is shown in Fig 14. The output value of MLP deviates greatly from the test value, and five test results are negative. The performance of XGBoost is significantly better than that of MLP, which is consistent with the overall trend of the test value. PSO_SVR is a support vector machine regression model based on particle swarm optimization. The distribution trend of its output value and test value is basically consistent, but it deviates greatly from the multiple test values, such as test data No. 2, No. 4, No. 5, and No. 19. NANEAT and ANEAT are derived from the models generated by evolutionary neural networks, and they both agree well with the test values. However, according to the data distribution, the approximation degree of ANEAT is better than NANEAT. Moreover, ANEAT’s predictive performance is better than that of the other three models.

The above comparative analysis shows that the data distribution of different methods on the test set is consistent with the evaluated values in Table 3. It shows the effectiveness of ANEAT in CGO prediction.

## 5 Discussion

This research aims to achieve high-precision and lightweight CGO prediction for the problems of small sample data and imbalanced distribution. It breaks through the limitations of the preset model architecture of traditional methods and realizes the collaborative optimization of structure and weights. The comparative experimental results show that ANEAT has the lowest *MAE* and *RMSE* on the test set. Moreover, the *EVAR* value of this method is 0.8972, which is higher than other methods and has the best predictive performance. It indicates the effectiveness of data augmentation and neuroevolution in this research.

The improvement of the ANEAT model in CGO predictive performance can be attributed to three main factors. First, unlike the raw data, the augmented data enriches the diversity of samples and improves the representation ability of the data. Data in the real world is often highly complex and diverse. However, due to the limitations of collection conditions, the number of high-risk CGO samples is insufficient, which leads to imbalanced data samples and inadequate diversity. In the experiment, the transfer function *T*(*i*, *j*) is applied to the same sample multiple times, enhancing the number and diversity of samples. It is beneficial for the model to learn the essential invariance of data under different transformations to maintain stable performance in actual scenarios. Secondly, Sparse PCA retains the critical information by optimizing the feature combination and eliminates features that have little impact on the results. For example, after Sparse PCA dimensionality reduction on the augmented data, the features of the data samples are denoted as f1-f8. These features constitute the initial genome code of the evolutionary neural network and avoid redundant hidden nodes and connections during evolution. It helps the model capture the intrinsic relationships of the data more accurately, thereby improving its prediction accuracy. Thirdly, neural evolutionary networks can adaptively adjust structures and parameters under a global search mechanism. This global optimization and flexible adaptability can obtain solutions closer to the global optimal when facing complex problems. Compared with traditional machine learning and deep learning, ANEAT reduces reliance on human experience and improves the automation level of model construction through natural evolution mechanisms. In the experiment, we set parameters such as fitness_threshold, pop_size, and evolutionary generations to start the model’s evolutionary training. As the population species evolves, the fitness gradually increases and tends to stabilize. The champion organism emerged, and we obtained the optimal neural network phenotype. The global optimization and adaptive adjustment of structure and parameters under the natural evolutionary mechanism may become an essential factor that makes ANEAT superior to other methods.

This research presents an innovative exploration of CGO prediction under insufficient samples and imbalanced distribution conditions. A principal finding from our research suggests that data augmentation and neuroevolution can significantly improve CGO predictive performance. Our research is a new exploration based on the CGO prediction of Sun (2019) [32] from different perspectives. Sun (2019) collected CGO feature data through the mining engineering collaborative big data cloud platform. The data they released has significantly contributed to the exploration and research of CGO intelligent prediction. They extracted the main feature factors through grey correlation analysis and constructed a CGO prediction model based on PSO_SVM. Unlike their research, we used Sparse PCA instead of grey correlation analysis to extract the main features. Moreover, the data augmentation with pointwise intensity transformation was performed on the raw data. In terms of model construction, Sun (2019) introduced PSO to optimize two parameters of SVM. ANEAT involves more parameters and optimizes the neural network structure and weights collaboratively. It has superior advantages in automated model construction and data-driven performance. In response to the lack of data information and imbalanced sample data, Tian et al. (2024) proposed a data augmentation method for fault diagnosis with class-imbalance problem. Their research demonstrates that the data augmentation methods can improve the learning effect in class-imbalance fault diagnosis [34]. This research is similar to ours in that they both use respective data augmentation techniques to improve data-driven performance. The effectiveness of the two experimental results is consistent. In addition, some scholars have applied evolutionary machine learning algorithms to cardiovascular disease risk prediction. They used a genetic algorithm to develop an easy-to-use model with high accuracy. Compared with traditional methods, this method greatly improves the ability to predict cardiovascular disease [35]. Their method and the evolutionary neural network used to construct the ANEAT model both reflect the evolutionary process of genome coding, mutation, and recombination. They are all clear and interpretable methods. The excellent performance of evolutionary algorithms has been revealed in both cardiovascular disease risk prediction and CGO prediction. The above researches illustrate the potential application value of data augmentation and evolutionary algorithms in risk prediction. Although the problem areas are different, the results show the effectiveness of data augmentation and neuroevolution in CGO prediction.

The method we proposed also has certain limitations. The advantages of classification or regression are not obvious on datasets with large samples and rich feature expressions. The main reason is that the large sample data already contains sufficient diversity, and further data augmentation may introduce redundant data. It does not contribute positively to the improvement of model performance. In addition, a large number of candidate neural evolutionary networks need to be evaluated on large datasets. This has certain limitations for its application on large datasets.

## 6 Conclusion

The ANEAT model proposed in this paper can better extract and express the sample features of CGO factors, optimize neural network structure, and improve the prediction efficiency and accuracy of CGO risks. Data augmentation gives gas characteristics, coal seam characteristics, and geological structure parameters more robust data representation capabilities. It enriches the number of samples and improves the data quality. The evolutionary neural network simulates the natural evolution mechanism of population species. The model evolves from the simple genome of a single species in the population, eliminating traditional machine learning or deep learning preset model architecture constraints. The ANEAT model realizes the collaborative optimization of structures and weights, and it completes the high-precision mapping of feature parameters and outburst risks with a lightweight network architecture. In model construction and evolution, population fitness changes and speciation results show that data augmentation can better guide the model to evolve in a beneficial direction, positively affecting model optimization. ANEAT has a lower mean absolute error and root mean square error, and the explainable variance is better than the other four models. In the future, we will further strengthen the interdisciplinary research between computer science and CGO disaster prevention and control. In addition, the application of ANEAT to CGO prediction, multi-objective optimization, and automatic machine learning in specific scenarios remains the content of follow-up exploration research.

## Supporting information

S1 Data (ZIP)
