# Supplementary material for: Coal and gas outburst prediction based on data augmentation and neuroevolution
Source: PLoS One. 2025 Feb 20;20(2):e0317461. doi: 10.1371/journal.pone.0317461 (PMC11841871; doi:10.1371/journal.pone.0317461)
Supplement: S1 Data — (ZIP) [file pone.0317461.s001.zip › description.docx]

**The raw data comes from the following references:**

Sun L. Application research on the coal and gas outburst prediction based on gray correlation analysis and PSO-SVM. M.Sc. Thesis, China University of Mining and Technology. 2019. Available from: https://d.wanfangdata.com.cn/thesis/ChJUaGVzaXNOZXdTMjAyNDAxMDkSCUQwMTY5Mjc4MhoIbW9vZzN5NGE%3D

**The meaning of each column of the data sample is:**

Column 1: Types of coal damage

Column 2: The initial velocity of a gas release

Column 3: Coal seam gas content

Column 4: Coal seam gas content

Column 5-7: Gas desorption index of drill cuttings k1

Column 8-10: Cuttings amount

Column 11: Distance from geological tectonic zone

Column 12: Buried depth

Column 13: Coal thickness

Column 14: Gas outburst risk
